# Production of Bovine Colostrum for Human Consumption to Improve Health

**DOI:** 10.3389/fphar.2021.796824

**Published:** 2022-01-03

**Authors:** Merve Kaplan, Ayşenur Arslan, Hatice Duman, Melda Karyelioğlu, Barış Baydemir, Bilgetekin Burak Günar, Merve Alkan, Ayşe Bayraktar, Halil İbrahim Tosun, Melih Ertürk, Günay Eskici, Rebbeca M. Duar, Bethany M. Henrick, Steven A. Frese, Sercan Karav

**Affiliations:** ^1^ Department of Molecular Biology and Genetics, Canakkale Onsekiz Mart University, Canakkale, Turkey; ^2^ Department of Coaching Education, Canakkale Onsekiz Mart University, Canakkale, Turkey; ^3^ Department of Physical Education and Sports Teaching, Canakkale Onsekiz Mart University, Canakkale, Turkey; ^4^ Uluova Dairy, Canakkale, Turkey; ^5^ Evolve Biosystems, Inc., Davis, CA, United States; ^6^ Department of Food Science and Technology, University of Nebraska Lincoln, Lincoln, NE, United States; ^7^ Department of Nutrition, University of Nevada Reno, Reno, NV, United States

**Keywords:** bovine colostrum, immunoglobulins, thermal processing, drying methods, nanotechnology, liposomal technology, human consumption

## Abstract

Colostrum contains all essential nutrients for the neonate during the first days of life, with impacts that continue far beyond these first days. Bovine colostrum has been used for human consumption due to the high concentrations of bioactive proteins, vitamins, minerals, growth factors, as well as free and conjugated oligosaccharides. Processes involved in the preparation of bovine colostrum for human consumption play a pivotal role in preserving and maintaining the activity of the bioactive molecules. As bovine colostrum is a multifunctional food that offers a myriad of benefits for human health, assessing the main processes used in preparing it with both advantages and disadvantages is a crucial point to discuss. We discuss major processes effects for colostrum production on the nutritional value, some advanced technologies to preserve processed bovine colostrum and the end-product forms consumed by humans whether as dairy products or dietary supplements.

## 1 Introduction

Colostrum is the first fluid secreted by mammals for the first few days after parturition (Marnila and Korhonen, 2002; Stelwagen et al., 2009; Godhia and Patel, 2013). This food provides the initial supply of vital nutrients for neonates and plays a crucial part in the nutrition, protection, development, and immunological defense of the newborn (Rasmussen et al., 2016; Juhl et al., 2018). In the absence or limited availability of human milk, bovine colostrum is widely used as an alternative source for infants (Li et al., 2017). As newborn calves do not have an active adaptive immune system of their own, the high concentration of antibodies in colostrum, immunoglobulins, have a major impact on priming the calf’s immune system. The small intestine of calves is permeable for the passive transfer of colostrum immunoglobulins through the intestinal wall for only a limited time. The potential for this passive transfer decreases in the first 6–12 h postnatal and becomes impermeable to immunoglobulins by about 24–48 h after birth. Therefore, it is vital to provide colostrum as soon as possible after birth to calves (Sangild, 2003; Baintner, 2007). Even though calves develop a gut closure that stops immunity transfer from mother, colostrum is produced in excess for several days (Marnila and Korhonen, 2002; Stelwagen et al., 2009; Godhia and Patel, 2013). Moreover, bovine colostrum comprises only 0.5% of a bovine’s milk output in a year, but this is far more than the calf’s requirements (Oyeniyi and Hunter, 1978; Scammell, 2001). For this reason, excess colostrum has a high potential for use in various industrial-scale applications (Alexieva et al., 2011).

Bovine colostrum is comprised of essential nutrients such as proteins, fats, vitamins, and minerals. Additionally, bovine colostrum contains high levels of bioactive compounds including oligosaccharides, immunoglobulins, lactoferrin, and lysozyme (Kehoe et al., 2007), which have well-characterized roles in providing passive immunity, antimicrobial protection, and shaping the development of the gastrointestinal system in the early life of calves (Playford et al., 2001; Elfstrand et al., 2002). In addition to bovine colostrum’s benefits on the health of the calves, it also improves human health in that it enhances the immune defense and gastrointestinal health of humans by its bioactive components (Arslan et al., 2021). The link between consumption of bovine colostrum by humans and the immunity defense has been investigated for long years to better understand bovine colostrum’s immunological activity. Several studies have already shown that bovine colostrum has been used in clinics and therapeutical applications (Godhia and Patel, 2013; Gephart and Weller, 2014; Dzik et al., 2017). Oral intake of bovine colostrum, for instance, impacts intestinal immunity, which is resulted from an increased concentration of natural killers, lymphocytes (Hurley and Theil, 2011; Wong et al., 2014). IgG in bovine colostrum is also able to alter innate and adaptive immunity by binding to pathogens and human Fcγ R receptors which leads to phagocytosis and defense (Hessell et al., 2007).

Bovine colostrum, furthermore, is considered to be an effective option for passive immunity with its rich IgG content against a serious disease. The vaccination of cows against disease-causing pathogens before taking their colostrum samples can enhance the specificity of IgG content. IgG derived from hyperimmune bovine, in turn, can bind to the virus directly and hinder the binding of pathogens to intestinal epithelial cells (Jawhara, 2020). Regarding severe acute respiratory syndrome coronavirus 2 (SARS-CoV-2), which causes a serious respiratory disease called coronavirus disease 19 (COVID-19) in humans, the specificity of IgG in colostrum against this virus may increase by the vaccination of cows and when people consume this hyperimmune colostrum, they may also be protected in short-term. In a clinical study, a human polyclonal IgG antibody which is derived from cattle vaccinated against MERS is considered safe and tolerable up to 50 mg/kg in healthy individuals (Luke et al., 2016; Beigel et al., 2018).

Bovine colostrum is also frequently preferred by athletes who do both endurance and resistance exercises since it offers high nutritional value. Athletes consider keeping themselves physically and physiologically at the highest level in a highly competitive environment. For this, in addition to nutrition programs, they mostly use a variety of supplements. Among these supplement products, bovine colostrum is also located a natural and healthy food source. Various studies have been shown that bovine colostrum impacts on the immune system (Pyne et al., 2015; Jones and Davison, 2019; Skarpańska-Stejnborn et al., 2020) and muscle development (Antonio et al., 2001) in athletes. Studies have shown that the use of colostrum in athletes is effective in hypertrophy, increase in muscle strength, and decrease in body fat (Antonio et al., 2001). Bovine colostrum, therefore, contributes to the prolongation of exercise duration by increasing the muscle buffering capacity during high-intensity exercises due to its rich IGF-1 content (Główka et al., 2019). For these reasons, bovine colostrum has been proposed as a way to improve human health in a variety of dietary supplements, dairy products, and nutraceuticals as a result of its bioactive potential. There are various preserved forms of bovine colostrum for human consumption as powder, liquid, tables, or capsules (Bartkiene et al., 2018; Silva et al., 2019). However, when considering the processes involved in preparing it for human consumption, the preservation and protection of the significant bioactive factors in bovine colostrum is critical (Phipps et al., 2016; Sotudeh et al., 2019). Thermal treatment and drying methods are some of the processes used to produce different forms of bovine colostrum, and each has advantages and disadvantages depending on the intended final application (Jay, 1992; Godden et al., 2006; Sotudeh et al., 2019). Therefore, the main aim of this review is to discuss the effects of thermal treatment and drying processes on the nutritional value of bovine colostrum on human consumption, as well as advanced technologies (e.g., nanotechnology and liposomal technology) to preserve processed bovine colostrum for human consumption. Moreover, end-products consumed by humans such as dietary supplements and dairy products are also discussed.

## 2 Thermal Treatment Methods

The preservation of high-quality colostrum that is free from microbial contamination is one of the main challenges to producing colostrum-based products for human consumption (Phipps et al., 2016; Sotudeh et al., 2018). There are several pathogens that cause contamination of colostrum, but contamination is principally caused by *Mycobacterium avium ssp. Paratuberculosis, Mycoplasma spp., Escherichia coli* (*E. coli*)*, and Salmonella spp.* (Streeter et al., 1995; González and Wilson, 2003; Houser et al., 2008). These pathogens often originate from the mammary glands and contaminate colostrum during milking, and can grow during storage, creating a potential risk for the final product (Fecteau et al., 2002; McGuirk and Collins, 2004). In calves, it may interfere with the passive absorption of colostrum antibodies, but in colostrum for human consumption, it can affects the protein and colostrum-based products’ quality (Houser et al., 2008). Therefore, effective elimination of pathogens requires thermal processing, most commonly pasteurization and sterilization (Jay, 1992), to ensure high-quality colostrum production (Sotudeh et al., 2018).

### 2.1 Pasteurization

Pasteurization is the heat-treatment process to destroy pathogenic microorganisms such as bacteria, protozoa, molds, and yeasts in beverages and foods (Jay, 1992). Pasteurization reduces the number of viable microbial cells rather than destroying all microorganisms (Elizondo-Salazar and Heinrichs, 2009). There are two pasteurization methods: high-temperature short-time (HTST) and low-temperature long-time (LTLT) pasteurization (Jay, 1992).

#### 2.1.1 High-Temperature Short-Time (HTST) Pasteurization

One of the most common pasteurization methods is HTST, also known as flash pasteurization or continuous pasteurization. In HTST, colostrum is circulated and heated up to 72°C (161°F) during 15 s (s) as it passes through heated coils (Jay, 1992). If the appropriate temperature is not reached during the first circle, the fluid is discharged back into the original tank, and the circulation process is repeated. These systems are designed to then rapidly cool the colostrum to feeding or storage temperatures (Elizondo-Salazar and Heinrichs, 2009). Several studies have shown that HTST processing on bovine colostrum is sufficient to destroy many common pathogens in raw milk, such as *Mycobacterium paratuberculosis (M. paratuberculosis), Salmonella enterica spp*., and *Mycoplasma spp.*, while others have shown that *M. paratuberculosis* is destroyed using HTST on bovine colostrum (Godden et al., 2003; Stabel et al., 2004). Stabel et al. (2004) showed that HTST pasteurization was sufficient to remove viable *M. paratuberculosis*. However, notable reductions in the quality of bovine colostrum were observed, including approximately 25% reduction in intact immunoglobulin G (IgG) concentration after HTST pasteurization. Additionally, the process significantly increases the viscosity of the fluid during or after the pasteurization process (Godden et al., 2003). Taken together, these studies suggest that HTST pasteurization is an efficient method of pathogen destruction while simultaneously ensuring the majority of bioactive molecules remain to produce high-quality colostrum-based products for human consumption (Elizondo-Salazar and Heinrichs, 2009). However, special considerations for the increase in viscosity are important for downstream applications.

Microfiltration is another process, which can be combined with HTST, as an alternative way to extend the shelf-life of dairy products (Al-Akoum et al., 2002). This process is increasingly used in the dairy industry to remove bacteria by membranes, which is also feasible in technique and economics. Microfiltration eliminates all pathogens from bovine colostrum or milk regardless of any denaturation of bioactive proteins or nutritional content (Hansen et al., 2020). It may also preserve the bioavailability of multifunctional components including peptides, antioxidants, and vitamins (Papadatos et al., 2003; Amelia and Barbano, 2013). Tetra Pak developed a process called Bactocatch which is the most common microfiltration to remove bacteria from the products. Membrane fouling is decreased significantly by a crossflow uniform transmembrane pressure in the bactocatch system (Sandblom, 1978). Microfiltered retentate and cream parts of dairy products are treated under 130°C for 4 s and combined with skimmed permeate which is almost bacteria free and the milk is processed with a minimum HTST. The bactocatch process reduces total bacteria by 2.8 logs in reports (Bindith et al., 1996; Hoffmann et al., 1996). In a study, bovine colostrum is treated by a membrane filtration process (pore size 0.1 μm). The process recovered at least 80% of the IgG and other minor whey proteins in the micro filtrate part (Piot M et al., 2004).

#### 2.1.2 Low-Temperature Long-Time (LTLT) Pasteurization

LTLT is another thermal treatment that is also known as Holder pasteurization or batch pasteurization. LTLT pasteurization is performed when a tank or batch of colostrum is heated up to the target temperature [63°C (145°F)], for a minimum of 30 min (Jay, 1992). LTLT pasteurizers should be equipped with an agitator for allowing to homogeneous heating throughout the entire batch of fluid. Following the 30 min heat treatment, batches are quickly cooled to feeding or storage temperature. One challenge with LTLT pasteurization is the batch size of the sample and the processing time. For instance, batch size impacts on the duration of pasteurization, when sizeable batches are used for pasteurization, the processing time may take several hours. Thus, some bacteria (e.g., some *Salmonella* spp.) may survive the process.

In contrast to HTST pasteurization, LTLT pasteurization of bovine colostrum was less effective at removing bacterial contaminants. In Meylan et al. (1996), colostrum samples were inoculated with *M. paratuberculosis* and then heated to 63°C for 30 min to test whether *M. paratuberculosis* could survive pasteurization. They also examined the effect of pasteurization on IgG concentrations. *M. paratuberculosis* was not eliminated by pasteurization, but its growth was slowed. The mean IgG concentration of unpasteurized and pasteurized ones were measured as 44.4 ± 30.3 g/L and 37.2 ± 23.8 g/L, respectively, representing a fall of 12.3 ± 8.7% IgG, which was not interpreted as a hindrance for successful passive transfer of immunity to calves (Meylan et al., 1996).

In a similar study by Godden et al. (2003), the efficacy of the commercial batch pasteurization method on the concentration of IgG content and the feeding features of bovine colostrum samples was investigated. The effect of pasteurization on IgG concentrations of serum samples of calves fed unpasteurized and pasteurized colostrum was also examined. In contrast to Meylan et al. (1996), the study showed that LTLT pasteurization (63°C, 30 min) declined IgG concentration by 58.5 and 23.6% for 95-L and 57-L batches, respectively. Pasteurization of 57-L batches produced high quality (vs. 95-L) and standard or mildly thickened bovine colostrum that could be used to feed calves. When pasteurization effects on serum IgG level was analyzed, a significantly lower serum IgG concentration was found in calves fed pasteurized colostrum, relative to unpasteurized colostrum. For example, serum IgG concentrations of 40 calves fed unpasteurized colostrum (19.1 g/L) were 9.4 g/L higher than serum IgG concentrations of 55 calves that are fed pasteurized colostrum (9.7 g/L) when calves were fed 2 L colostrum at the first nutrition time. In contrast, serum IgG levels between eight calves fed unpasteurized bovine colostrum (16.1 g/L) and 20 calves fed pasteurized bovine colostrum (13.5 g/L) did not differ if calves fed 4 L at the first feeding. The study was concluded that batch pasteurization of colostrum would help preserve and maintain colostrum (Godden et al., 2003). However, Johnson et al. (2007) reported raised IgG absorption efficiency and higher IgG level of serum samples from calves fed colostrum pasteurized for longer than 30 min. Colostrum pasteurized at 60°C, 60 min showed higher level of serum total protein (TP) and IgG, also efficiency of IgG absorption was higher in calves fed pasteurized colostrum (TP = 6.3 mg/dl; IgG = 22.3 mg/ml; apparent efficiency of absorption = 35.6%) in comparison to calves fed unpasteurized colostrum (TP = 5.9 mg/dl; IgG = 18.1 mg/ml; apparent efficiency of absorption = 26.1%). There was no significant difference found in serum Ig function and serum concentrations of IgA, IgM, vitamin E, vitamin A, cholesterol, *β*-carotene between fed pasteurized vs. unpasteurized colostrum at 24 h of age. Thus, the batch pasteurization of colostrum at 60°C, 60 min caused the reduced bacteria concentration in colostrum with preserving the colostrum and serum IgG concentrations (Johnson et al., 2007).

While 30 min pasteurization is the minimum for Holder pasteurization, McMartin et al. (2006) aimed to identify optimum temperature and process time of pasteurization to ensure optimal IgG concentration, the viscosity of bovine colostrum, etc., For these purposes, 50 ml bovine colostrum samples from six batches were heated at 59, 60, 61, 62, and 63°C in A Rapid Visco Analyzer (RVA). According to the results, there were no significant differences in viscosity and IgG concentrations between unpasteurized colostrum samples (73.4 ± 26.5 mg/ml) and pasteurized colostrum samples (74.5 ± 24.3 mg/ml) after colostrum samples were heated to 60°C in a RVA for 120 min. On the other hand, 50 ml colostrum samples from 30 unique batches were heated for 120 min at 60 and 63°C in a RVA to confirm the preliminary results. The second experiment showed pasteurization of colostrum at 63°C for 120 min resulted in a 34% decline in IgG level and a 33% increment in viscosity of colostrum samples (McMartin et al., 2006), suggesting that batch-to-batch variation may influence some of the findings.

In another study of S. Godden et al. (2006), bovine colostrum samples from 30-L batches were firstly inoculated with *M. bovis* (10^8^ CFU/ml), *Listeria monocytogenes* (10^6^ CFU/ml), *E. coli* O157:H7 (10^6^ CFU/ml), *Salmonella enteritidis (S. enteritidis)* (106 CFU/ml), and *Mycobacterium avium subsp.* paratuberculosis (Map; 10^3^ CFU/ml) to describe the effects of pasteurization on IgG concentration of bovine colostrum and investigate the optimum duration of heating to eliminate pathogens. After inoculation, samples were heated up to 60°C, 120 min. The subsamples of colostrum at 15 min intervals throughout the pasteurization were collected to analyze bacterial culture, measure IgG level (g/L), and the activity of antibodies [log_2_ (bovine viral diarrhea virus type 1 serum neutralization titer)]. As in previous studies, there was no significant difference found in IgG concentration of colostrum samples (pre: 60.5 g/L; post: 59.1 g/L) after heating colostrum to 60°C at least 120 min and no effect found in pasteurization process on mean log_2_ bovine viral diarrhea virus type 1 serum neutralization titer (pre = 12.3; post = 12.0). Another important result of this study; after pasteurization of colostrum at 60°C for 30 min, some pathogens such as *M. bovis, L. monocytogenes, E. coli O157:H7*, and *S. enteritidis* were not detected in samples. *M. paratuberculosis* was not observed when the colostrum was heated to 60°C for 60 min. It was concluded that heating of colostrum to 60°C for 60 min was enough to reduce the concentration of pathogens (Godden et al., 2006). When taken together with the findings of Meylan et al. (1996), the minimum time for Holder pasteurization may not be sufficient for efficient elimination of pathogens but extending this time to 60 min may improve the effect without significant losses to the bioactive potential of colostrum.

Similarly, Donahue et al. (2012) investigate the effect of pasteurization at 60°C - 60 min on total bacteria counts and IgG concentration of bovine colostrum. It was found that the pasteurization process reduced colostrum total plate counts (−2.25 log_10_) and coliform counts (−2.49 log_10_) but did not significantly change colostrum IgG concentration (Donahue et al., 2012). These results are consistent with the study by S. M. Godden et al. (2012). Godden et al. (2012) performed a randomized controlled clinical trial using 1,071 calves from six commercial dairy farms. The serum IgG concentrations were higher in calves that were fed pasteurized colostrum (18.0 ± 1.5 mg/ml) compared with calves fed unpasteurized colostrum (15.4 ± 1.5 mg/ml). In addition, calves fed pasteurized colostrum were at lower risk for illness, which was attributed to the considerable decline in colostrum total coliform count (Godden et al., 2012).

In a different study investigating the effect of pasteurized colostrum (60°C, 60 min) feeding on the colonization of microorganisms in the neonatal calves’ small intestine, Malmuthuge et al. (2015) assigned three treatment groups including feeding fresh colostrum, feeding pasteurized colostrum, and control group. The total microbial colonization in the small intestine of calves was analyzed using quantitative real time-PCR and it was concluded that there was an increase in total bacterial colonization in the small intestine of calves, whether they were fed with fresh or pasteurized colostrum. In contrast, *Lactobacillus* was found in lower level in the small intestine of the pasteurized colostrum-fed group than in the control group. However, *Bifidobacterium* was detected 3.2 and 5.2-fold higher in the small intestine of feeding with pasteurized colostrum groups than the feeding with fresh colostrum and control groups. In addition to these, the colonization of *E. coli* was significantly lower in the small intestine of feeding with pasteurized colostrum group (Malmuthuge et al., 2015).

Elsohaby et al. (2015) studied the impact of different pasteurization processes (60°C and 63°C each for either 30 or 60 min) on IgG concentration of colostrum samples. Infrared (IR) spectroscopy, the reference radial immunodiffusion (RID) assay, digital and optical refractometers were used to quantify IgG concentration of colostrum. The average RID-IgG concentration of unheated and heat-treated colostrum samples was determined as 45.6 g/L and 30–48 g/L, respectively. So, there was no difference between unheated and heat-treated colostrum concentration at 60°C for 30- and 60-min, whereas the concentration of RID-IgG decreased to 31.1 and 30 g/L, after heat treatment at 63°C for 30 and 60 min, respectively. On the other hand, the average IR-IgG concentration of unheated and heat-treated colostrum samples was measured as 44.7 g/L and 37.7–42.5 g/L, respectively and the average IR-IgG concentration of unheated and heat-treated colostrum samples regardless of temperature and time were similar. The unheated and heat-treated colostrum IgG concentrations’ Brix scores were also analyzed, and no difference was found between these groups regardless of temperature and time. It was concluded that radial immunodiffusion, IR spectroscopy, and Brix refractometers could be used for measuring IgG concentration of colostrum after heat treatment at 60°C, but the decrease of colostral IgG concentration after heat treatment at 63°C could not be detected by using these methods (Elsohaby et al., 2015).

Apart from similar studies about the pasteurization effect on bovine colostrum IgG, Rafiei et al. (2019) investigated the impact of feeding pasteurized colostrum on the neonatal dairy calves’ health and performance. Calves fed with pasteurized colostrum showed lower diarrhea, and pneumonia symptoms, whereas no differences in skeletal growth measurements except body barrels were observed. IgG concentration, total plate count, and passive transfer of immunity after heat treatment of colostrum at 60°C for 30 min were analyzed and there was no difference in the IgG concentration of colostrum between pasteurized and unpasteurized colostrum (57.6 mg/ml, 60.6 mg/ml, respectively). According to the results of the total plate count experiments, the pasteurized colostrum samples have lower colostrum bacterial concentrations (-2.01 CFU/ml) (Rafiei et al., 2019). Similar studies by Armengol and Fraile, (2016) concluded that feeding calves with pasteurized colostrum and milk (60°C for 60 min, 63°C, 30 min) decreased morbidity and mortality rates by 9.8 and 3.7%, respectively. The increment in bovine respiratory disease morbidity in the first year of life and diarrhea in the first 180 days of life was observed in calves who were not fed pasteurized colostrum (Armengol and Fraile, 2016).

Similar to previous studies, Hesami et al. (2020) studied the impact of different pasteurization temperatures and duration such as 60°C for 30, 60, and 90 min respectively on IgG concentration of bovine colostrum. The feeding pasteurized bovine colostrum effect on the health and performance of Holstein calves were also evaluated in this study. IgG concentrations of untreated or pasteurized colostrum at 60°C for either 30, 60, or 90 min was found to be 67.63 ± 0.08, 66.17 ± 0.08, 63.07 ± 0.08, and 59.53 ± 0.09 g/L, respectively. There was not any significant difference in the concentration of fat, protein, and ratio between fat and protein after pasteurization. It was observed that the pH of heat-treated colostrum samples increased from 6.77 ± 0.003 to 6.79 ± 0.003 but the difference was not significant statistically (*p* > 0.05). In conclusion, calves fed pasteurized colostrum had a better overall health status, whereas calves fed with unpasteurized colostrum had a lower prevalence of diarrhea-induced pathogens. During lactation, calves’ health, growth characteristics, and physical performance were improved by feeding pasteurized colostrum (Hesami et al., 2020).

### 2.2 Sterilization

Sterilization is another thermal treatment process of heating samples for the destruction of all viable organisms. Sterilization can be achieved using several ways such as heating, ionizing radiation, high hydrostatic pressure, photodynamic effects, or other techniques. Conventional sterilization methods cause irreversible inactivation or destruction of crucial components of the microorganisms, even highly resistant bacterial spores. The ultra-high temperature process is also called UHT, one of the common methods of the sterilization process (Kumbár et al., 2015; Doyle, 2019).

#### 2.2.1 Ultra-High Temperature (UHT)

The ultra-high temperature (UHT) process is the sterilization of dairy products at 135°C (275°F) for an extremely short period (around 2–5 s) to extend the shelf-life at room temperature. UHT treatment eliminates all viable organisms, including spores, but destroys several important components of bovine colostrum. Pasteurized bovine colostrum samples result in mostly a 15–35% reduction of IgG concentration of colostrum, while UHT treated samples almost no functional concentrations of IgG (Li-Chan et al., 1995). On the other hand, the UHT process has basic features such as its ability to process colostrum continuously, aseptic handling of the products, and stability at room temperature for up to 8 weeks without any changes (Jay, 1992).

## 3 Drying Methods

**FIGURE 1:**
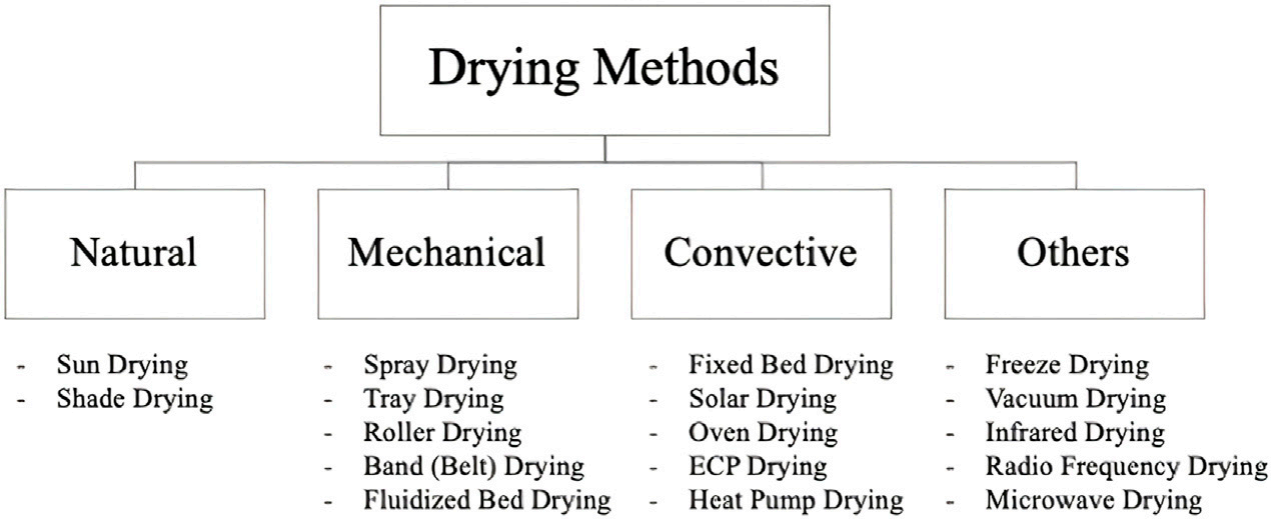
Drying methods.

Drying methods, as thermal treatment methods, are used in the food industry, especially in the food preservation process after pasteurization. There are many studies describing processes for drying, and they are critical for the food industry. Drying facilitates long storage periods and enables the processing of colostrum without any loss of its components (Guiné, 2018). During the drying process, the water activity in food products is lowered to slow microorganism growth and the speed of chemical reactions that may degrade colostrum quality. In addition, the reduced weight of dried, powdered products and long shelf stability reduces the costs and product packaging difficulties, storage, transportation and distribution of the products (Barbosa-Canovas et al., 2005). Powder products manufactured by drying methods have several advantages, for instance, special storage conditions are not required, and less space is required. Powder products are also good food reserves for emergency situations, and they are suitable to develop new food products (Schuck et al., 2016) There are a variety of drying methods from Sun drying to freeze drying, and spray drying processes to preserve food products (Figure 1). Spray drying and freeze drying methods are the most common methods favored in the dairy industry (Chelack et al., 1993; Stewart et al., 2005; McMartin et al., 2019).

### 3.1 Spray Drying vs. Freeze Drying

Spray drying is a technique for producing dried powdered products based on the transformation of liquid state into a dry powdered form. In the dairy industry, spray drying is the most used technique for the manufacturing of dairy powders and some ingredients due to its low cost and wide equipment availability. The feed, which can be a solution or suspension, is sprayed into a hot dryer chamber to rapidly remove moisture. The final product is in the form of powders, agglomerates, or granules (Borad et al., 2007). The spray drying process can produce spherical shape particles with a certain particle size distribution. It has a short drying time; therefore, it is applicable to drying heat-sensitive materials. Spray drying is mainly preferred to process milk (Oakley, 1997; Schuck et al., 2016). However, spray drying of colostrum causes significantly decreased concentrations of bioactive proteins such as transforming growth factor β1 and β2, and raised protein aggregation (Støy et al., 2016). Even though it is relatively cheaper and faster, changes in morphology and particle size, aroma loss, and thermal decomposition are major disadvantages of spray drying of milk products (Gharsallaoui et al., 2007), and the loss of functional proteins in colostrum also complicates the use of spray drying for colostrum, in particular.

Freeze drying is another drying method that includes two steps. First, the product is frozen, and the suspension medium is crystallized at a low temperature. Then, the fluid is sublimed from the solid-state through the vapor phase under reduced pressure (Ciurzyńska and Lenart, 2011). Freeze dried products retain their original shape and texture, and this procedure minimizes nutrient loss due to its low processing temperature and rapid transition of frozen material from hydrate to anhydrous. Therefore, it is the most favored dehydration technique for heat-sensitive biological materials (Borad et al., 2007). Freeze drying is frequently preferred in the biotechnology sector and freeze-dried products can be stored for more than a year without loss of their biological properties (Ciurzyńska and Lenart, 2011).

Spray drying and freeze drying have some advantages such as protection of elements, which are sensitive to high temperatures, against degradation. However, freeze drying of bovine colostrum is more effective in preserving bioactivity, especially of immunoglobulins, when compared to spray drying (Elfstrand et al., 2002). Whether spray or freeze dried, colostrum powders can be used as a dietary supplement or as ingredients in new products, and evaluations of freeze and spray drying methods in terms of production speed, cost, and energy use found that while the production rate of spray drying is higher and has an overall lower cost, freeze drying is more efficient and protects immunoglobulins in colostrum better than spray drying (Chelack et al., 1993; Borad et al., 2019). Elfstrand et al. (2002) investigated the effect of such dairy processing treatments as heat treatment, ultrafiltration, and freeze drying, on the recovery of Igs (IgG, IgA, and IgM), growth factors (TGF-β2 and IGF-1) in bovine colostrum. The 30 and 25% fall in IgG and IgA, respectively, were noted in colostrum concentrate after pasteurization and freeze-drying processes compared to untreated colostrum. According to results, pasteurization caused 33% decrease of IGF-1, while a further 30% reduction was observed in freeze drying. These results indicated that the bioactive components of the bovine colostrum decreased gradually by filtration, heat treatment, and freeze drying processes (Elfstrand et al., 2002). Sotudeh et al. (2018) examined the impact of spray drying, freeze drying, and pasteurization techniques on bacterial loads and the IgG concentration of first milking postpartum bovine colostrum. All treatment groups showed an impact on the reduction of standard plate count, *E. coli* count, and total coliform count, while the spray drying, and freeze-drying were noticeably efficient at reducing microbial loads. Additionally, a reduction in IgG level was observed in all treatment groups, but a lower decrease was noted in freeze dried bovine colostrum in comparison to spray dried and pasteurized bovine colostrum. These results indicated that freeze drying and spray drying processes are functional for enhancement of the shelf-life and quality of bovine colostrum as compared to pasteurization (Sotudeh et al., 2018).

The digestibility of protein, as well as the protein structure, can be affected by heat during processing. Different drying process conditions lead to various effects on protein digestion (van Lieshout et al., 2020). Altered digestion due to modifications of proteins may affect the physiological effects of dairy proteins to the consumer. The gastrointestinal tract and the immune system can also be affected by the differently sized peptides, and various chemical modifications of these sequences (Nowak-Wegrzyn and Fiocchi, 2009). The use of spray drying in the preparation of colostrum powder is limited, as it may cause heat-induced damage to colostrum proteins for example, causing disruption of key bioactive proteins such as lactoferrin and a loss in immunoglobulins (Bar et al., 2010; Abd El-Fattah et al., 2014; Borad et al., 2019). However, high costs in production, longer processing period, and scaling difficulties the freeze drying has limited some applications in the industry. Spray drying of colostrum generally costs 2.5 times less than freeze drying and commercial equipment is readily available (Chelack et al., 1993). Although excessive temperatures are avoided in spray drying to maintain immunoglobulin function in bovine colostrum products, it can cause the growth of pathogens and spoilage microorganisms. Therefore, spray drying conditions should be optimized for evaluating the thermal protection impact and to control microbial burdens with safety standards (Borad et al., 2019).

## 4 Advanced Technologies to Preserve Processed Colostrum for Human Consumption

Nanotechnology and liposomal technology are used to prepare bovine colostrum for human consumption with numerous advantages. Nanotechnology is a study of structures and materials on an ultra-small scale, and it has various applications in many fields from dairy industry to medicine (Chellaram et al., 2014). The nanotechnology principle is based on the modification of physicochemical properties of materials when they are reduced to the nano dimension (particles approximately 1 micron in size) (Keck et al., 2008). It provides several opportunities for the development of novel products and the expansion of food system applications including nutraceuticals, functional foods, bioactive substances, and pharmaceuticals, etc. (Samal, 2017). Even more, nanotechnology can be used to detect food pathogens, which are indicators of food quality and safety (Bott et al., 2014). This technology is increasingly used in the food market due to its advantages such as improving shelf life of products, flavor, and texture of foods. (Bajpai et al., 2012). Nanoencapsulation, which is nanometer encapsulation using films, nano dispersions, or layers, is a novel area of nanotechnology. The capsule derived by nanoencapsulation technology protects the food or active ingredients at the nanoscale. The key advantage of nanoencapsulation is the increased homogeneity, improved encapsulation efficacy as well as enhanced physical and chemical properties (Sanguansri and Augustin, 2006; Khare and Vasisht, 2014).

In the dairy industry, several bioactive proteins including whey proteins are preserved to be used as nutritional supplements. However, since most proteins are fragile, their stabilization is a crucial requirement for many applications. Nanoencapsulation technology encapsulates those bioactive proteins into nanometer-sized vesicles to prevent any denaturation or degradation that can result from proteolysis and/or dilution effects (Srinivas et al., 2010). Nanoencapsulation technology not only preserves peptide stability of proteins in dairy products, but also improves bioactive peptide delivery to target tissues (Dziuba and Dziuba, 2014). This technology provides significant advantages to avoid instability problems in the preservation of bovine colostrum and its bioactive proteins. The stability of bovine colostrum proteins, such as IgG, is crucial to preserve the bioactive potential of bovine colostrum. Bovine colostrum IgG encapsulated by 0.5% (w/v) of Tween 80, sucrose stearate, or soy protein showed increased stability of free IgG against the pH 12.0 and 2.0 environments by 33–62% and 21–56%, respectively, based on the emulsifier (Chen et al., 1999).

Liposomal technology, which is widely preferred in the pharmaceutical, cosmetic, and dairy industries, and genetic engineering, is another commonly used encapsulation method (Lipowsky and Sackmann, 1995; Jesorka and Orwar, 2008; Mozafari et al., 2008). This advanced technology uses a liposome, which is a spherical vesicle including at least one lipid bilayer and phospholipids. Liposomes can have surface ligands to ensure the binding of various ligands. These spherical vesicles can be used in many applications due to their independent biological activity and improved efficacy of encapsulated content (Umnova, 2010).

One of the liposomal technology applications is to prepare functional additives as food products for various therapeutic applications. Liposomal technology has been used to preserve bovine colostrum and increase its bioavailability for human health (Superti, 2020; Subramanian, 2021). The delivery of colostrum through the body with the reconstituted membrane phospholipids is far more effective in comparison to the colostrum without liposomal encapsulation. Furthermore, a preliminary study using liposomal encapsulated colostrum supplementation (20 g of colostrum per day) increased the bone-free lean body mass, whereas whey supplementation did not cause any increase in lean body mass (Antonio et al., 2001). With this advanced liposomal delivery system, these results indicated that there was a remarkable increase in the bioavailability of bovine colostrum. The embedding of active components into liposomes has some advantages such as enhanced bioavailability of components, controlled release of active component through the body, and prevention from any environmental factors such as temperature, oxygen, pH etc., (Mozafari et al., 2008). Thus, nanoencapsulation and liposomal technology may offer significant advantages for preserving a food product and enhancing its therapeutic activity in many food applications.

## 5 Forms Consumed by Humans (e.g., Liquid, Cheese, Powder, Pill/Tablet)

In recent years, bovine colostrum has been used for human health in various dietary supplements, nutraceuticals, or in traditional medicine due to its rich nutritional content that claims to block pathogens, improve body condition, and enhance gastrointestinal health (Bartkiene et al., 2018; Juhl et al., 2018; Silva et al., 2019). Several processing treatments (thermal treatment, drying, and others) are used to prepare bovine colostrum for human consumption. End-products through these processes can include liquid colostrum, a preserved dairy product, infant formula, pills, tablets, liquid, or as a powder supplement. Additionally, advanced technologies such as nanoencapsulation and liposomal technology are recently preferred to manufacture bovine colostrum for human health products (Chellaram et al., 2014).

### 5.1 Forms of Bovine Colostrum in Dairy Products

Liquid bovine colostrum is currently used as an ingredient in some dairy products such as yogurt, cheese, or kefir. The production of dairy foods with addition of bovine colostrum is the focus of several studies to ensure its suitability for human consumption (Table 1). The addition of bovine colostrum into dairy products is thought to provide an improved and healthier product for humans. In the context of yogurt production, the bovine colostrum addition represented great sensory acceptance (Ayar et al., 2016; Abdel-Ghany and Zaki, 2018). Abdel-Ghany and Zaki, (2018) and the addition of bovine colostrum increased the total protein content, total solids, IgG, lactoferrin, and minerals compared to typical yogurt (Abdel-Ghany and Zaki, 2018). Similarly, Saalfeld et al. (2008) showed sensory acceptance for milk-based beverages and butter including bovine colostrum silage, which is a kind of anaerobic fermentation methodology for excessive colostrum used for allowing milk replacement for calves (Saalfeld et al., 2008). Other traditional dairy products have also been evaluated for the addition of colostrum. Poonia and Dabur, (2012) evaluated the addition of bovine colostrum in a traditional Indian dessert known as khess (Poonia and Dabur, 2012). Das and Seth, (2017) also studied curd samples fortified with the bovine colostrum whey addition. In addition to these dairy products, bovine colostrum has also been used in cheese. For instance, a baked cheese, which is known as leipajuusto, is traditionally produced in Finland from bovine colostrum or reindeer milk. A sweet cheese called junnu is also made with bovine colostrum in southern states of India (Das and Seth, 2017).

**TABLE 1:** Studies evaluating the addition of bovine colostrum in dairy production.

Product	References
Yogurt	Ahmadi (2011)
Yogurt and kefir	Ahmadi (2011)
Fermented milk	Nazir et al. (2018)
Curd	Das and Seth (2017)
Khees	Poonia and Dabur (2012)
Ice cream	Mouton and Aryana (2015)
Milk-based beverages and butter	Saalfeld et al. (2008)

### 5.2 Forms of Bovine Colostrum in Dietary Supplements

There is a noticeable increment in the number of people who consume bovine colostrum supplements with the desire to improve health (Arslan et al., 2021; Davison, 2021; Playford and Weiser, 2021). Bovine colostrum supplements can be used in several forms such as powder, liquid, tablets, or pills. The colostrum products market has different tablets containing dried colostrum, colostrum-based drinks, or dried colostrum powders. Drying processes to produce bovine colostrum supplements are appropriate for hindering protein denaturation. Specialized low heat spray drying or freeze drying are also favored to prevent these types of denaturation (Chelack et al., 1993; Stewart et al., 2005; McMartin et al., 2019).

Bovine colostrum use as a dietary supplement improves human health and wellbeing (Playford et al., 2001; Silva et al., 2019). This supplement is especially taken by athletes to improve body functions and high-intensity exercise (Mero et al., 1997, 2002). Current studies showed that the bovine colostrum supplement has some positive effects such as reducing exercise-induced muscle damage and inflammation (Kotsis et al., 2018; Jones et al., 2019). Additionally, bovine colostrum is used in the prevention against diarrhea, for improved immune system function, the treatment of AIDS-related gastrointestinal diseases, and to improve gut health (Florén et al., 2006; Patel and Rana, 2006). Further, there is some evidence that bovine colostrum helps in immune modulation, maintenance of gastrointestinal mucosal integrity, and/or promoting tissue repair (Table 2).

**TABLE 2:** Different bovine colostrum forms used in some types of diseases.

Disease	Colostrum form	Effects	Dose	References
Gastrointestinal injury by non-steroidal anti-inflammatory drugs	Powder colostrum in tablets/capsules	IGF and TGF-B linked with analgesic activity	125 ml daily	Playford et al. (2001)
Diabetes delayed injury healing by rise in the level of blood glucose	Colostrum topical cream/powder colostrum in tablets/capsules	Reduction of blood glucose	10 g daily	Kim et al. (2009)
Inflammation induced for HIV patients	Colostrum powder in the form of tablets/capsules or liquid colostrum	Mucosal integrity, tissue repair, antimicrobial actions	10 g daily	Kaducu et al. (2011)
Ultraviolet B induced photodamage	Colostrum topical cream	Preventing damage to skin	10 g daily	Murata et al. (2014)
Gut permeability problems	Colostrum powder	Preventing gut stability	20 g daily	Marchbank et al. (2011)
Inflammation in colon	Colostrum liquid	Decreasing in symptoms	100 ml daily	(Khan et al., 2002
Acute infectious diarrhea	Colostrum powder	Diarrhea and lower frequency of vomiting	3 g sachet with 50 ml water	(Menchetti et al., 2016; Barakat et al., 2019)

## 6 Conclusion

Food products are processed to ensure both high quality and safety while preserving their nutritional content and extending shelf-life. Practically, processing aims to destroy microbes associated with spoilage and disease, the inhibition of native enzymes, minimization of the deterioration rate of the product, and prevention of nutrients in the food. Bovine colostrum has been used in several food products and dietary supplements due to its rich nutritional content, which may confer health benefits for humans. Various processes (e.g., pasteurization, sterilization, spray drying, and freeze drying) are used to prepare bovine colostrum as appropriate for human consumption. Such processes have both advantages and disadvantages, whereas their common aim is to preserve colostrum quality associated with bioactive proteins, such as IgG, for human health. Therefore, ensuring stability of these proteins is a necessity to preserve the beneficial effect of bovine colostrum.

Thermal treatments including HTST, LTLT pasteurization, and UHT sterilization that have been investigated by several researchers for the development of bovine colostrum-based products without reducing its quality and IgG level. Thermal denaturation temperatures of different protein fractions of bovine colostrum range from 62 to 78°C whereas the immunoglobulins begin to denature at 72°C and they are fully denatured at 89°C. According to some studies, HTST pasteurization is an effective method to destroy some pathogens in colostrum but resulted in a reduction in colostrum immunoglobulins and unacceptable feeding characteristics, namely an increase in viscosity. LTLT pasteurization significantly eliminated several pathogens and reduced total bacteria and coliform counts in colostrum, while preserving IgG concentrations and the nutritional composition of colostrum. In the UHT sterilization process, bioactive proteins are almost entirely degraded due to their sensitive structure, though sterilization otherwise extends shelf-life for bovine colostrum products and eliminates all viable microorganisms. Sterilization can be tolerated in processing of milk and milk-based products, but this process causes considerably decreased bovine colostrum quality. As a result, typical pasteurization methods can be practical to maintain quality and feeding characteristics of bovine colostrum. Drying methods are also used to preserve therapeutic potential of colostrum immunoglobulins. Freeze drying is the favored dehydration method for heat-sensitive biological samples due to its low processing temperature. This method is useful for minimizing nutrient loss and obtaining a high quality final product. In the context of bovine colostrum, some research indicates that freeze-drying preserves bioactive proteins, enhancing the quality and shelf-life of bovine colostrum as compared to spray drying.

In addition to these processes, some advanced technologies such as nanotechnology and liposomal technology use encapsulation processes to deliver bovine colostrum in a finished product. Various commercial forms of bovine colostrum as end-products are developed with different processes and used for different purposes, each choosing the advantages of a particular processing scheme for the desired end use. The interest in using bovine colostrum in some food products is increasing and it is considered to be a novel functional food.
